# Classification status of drugs generally used for self-medication in children: a targeted review of regulatory documents

**DOI:** 10.1080/20523211.2026.2619303

**Published:** 2026-02-02

**Authors:** Rizqi Dinni Fauzia, Hardika Aditama, Su Myat Thin, Noppadon Adjimatera, Puree Ananthachoti

**Affiliations:** a Department of Social and Administrative Pharmacy, Faculty of Pharmaceutical Sciences, Chulalongkorn University, Bangkok, Thailand; b Department of Pharmaceutics, Faculty of Pharmacy, Universitas Gadjah Mada, Yogyakarta, Indonesia; c Thailand Self-Medication Industry Association, Bangkok, Thailand

**Keywords:** Self-medication, drug classification status, children, paediatric medicines

## Abstract

**Background:**

Self-medication given by parents or caregivers is a common practice among children. Several frequently used drugs are often administered without professional supervision. This study compares regulatory classifications of common paediatric self-medication drugs across ten countries, aiming to uncover trends based on product type and national policies.

**Methods:**

Data were primarily collected from official drug regulatory agency websites and supplemented from other relevant sources. Twelve widely used drugs were reviewed, by focusing on classification status, approved indications, and paediatric age restrictions. Content and comparative analyses were conducted.

**Results:**

The findings revealed that drugs indicated for fever, pain, mucolytic effects, and non-sedating antihistamines were mostly classified as non-prescription. For drugs with the same active ingredient, but different strengths or dosage forms, compared within the same country, the classification status generally remained unchanged, even across different paediatric age groups. Drugs for asthma and topical corticosteroids were likely to be classified as prescription drugs. In some countries, such as the United Kingdom, Australia, and Singapore, the same drug preparation had different classification statuses which were based on factors such as approved indication and pack size. Additionally, Singapore and Indonesia implement prescription-exemption systems. These allow supply of certain prescription drugs without a prescription under specific conditions.

**Conclusion:**

Commonly used drugs for paediatric self-medication are classified as both non-prescription and prescription. This reflects diverse regulatory approaches across countries. Regulators play a key role in ensuring safe use of self-medication in children. Lack of harmonisation address a critical need for globally consistent paediatric drug classification scheme. They empower consumers through mechanisms such as clear, accessible patient information leaflets and other educational tools.

## Background

Self-medication (SM) is routinely used for treating minor and self-limiting illnesses. SM includes using both non-prescription and leftover prescription drugs (Bowen et al., 2000; Lifshitz et al., 2020; Shehnaz et al., 2014). SM prevalence in adults has ranged from 31% to 95% (Ghasemyani et al., 2022; Limaye et al., 2017; Tarciuc et al., 2022). Additionally, it is notably high in children, which has ranged between 25% and 96% (Ahmed et al., 2021; Ge et al., 2021; Gohar et al., 2017; Tarciuc et al., 2022; Yuan et al., 2022). Routine drugs applied for SM in children comprise cough and cold treatments, analgesics and antihistamines (Anderson et al., 2013; Gualano et al., 2015; Nydert et al., 2011; Shehnaz et al., 2014; Sturkenboom et al., 2008). Prior studies have demonstrated SM’s prevalence across countries with differing socioeconomic status (Bi et al., 2023; Gualano et al., 2015; Pereira et al., 2007; Shehnaz et al., 2014).

SM’s relevance is perceptible from diverse standpoints. For the populace, SM provides a swift and expedient treatment for minor health concerns not necessitating medical consultation, which aids timesaving and reducing absenteeism from work or school (AESGP Foundation, 2004; Bennadi, 2014; Janatolmakan et al., 2022). From the clinician standpoint, SM can mitigate healthcare facility usage by diminishing patient volume for minor ailments, enabling clinicians to focus on more severe cases (Bennadi, 2014; Ferner, 1994; Hughes et al., 2001). Finally, from a government standpoint, extensive SM usage may diminish public healthcare disbursements by mitigating treatments for non-critical conditions, thereby releasing resources for more serious healthcare conditions (Bell et al., 2016; Blenkinsopp & Bradley, 1996; Centers for Medicare & Medicaid Services, 2022; 2024; Hughes et al., 2001).

Multifactorial SM practices are affected by individual demographics, healthcare system structure, and regulatory policy regulating drug use. Individually, factors such as elder, higher education, and previous exposure to illnesses significantly escalate the prospect of participating in SM practices (Alonso-Castro et al., 2022; Esan et al., 2018; Fadare & Tamuno, 2011; Ge et al., 2021; Hughes et al., 2001). The organisation of the healthcare system makes access to services difficult, which often motivates individuals to practice SM as a more reasonable and timely alternative (Bennadi, 2014). Moreover, drug regulatory policies pivotally impact SM practices, with factors such as drug classification, reclassification from prescription to non-prescription status, and the insufficient enforcement of prescription drug dispensing regulations all fostering to SM practices (Gauld et al., 2019; Ruiz, 2010).

Leelavanich et al. (2020) analysed drug classification across eight nations: Canada, Japan, Singapore, the United States, the United Kingdom, Malaysia, the Philippines, and Thailand. Their research identified three consistent criteria for classifying drugs as prescription or non-prescription: disease characteristics, drug safety profiles, and other characteristics such as pack size, dosage form, or unique administration requirements. Regardless of these shared guidelines, notable classification inconsistencies materialised between countries. For example, nicotine patches were prescription-only in Japan, behind the counter in Singapore and Malaysia, on open shelves in Thailand, and on general sale lists in Canada, the USA, and the UK. Comparably, Ibuprofen 400 mg was classified as prescription in the USA, behind the counter in Malaysia, the Philippines, and Thailand, but on general sale in Canada.

While SM in children is common and has significant implications for clinical practice and regulatory policy, its regulatory landscape is understudied. A comparative analysis of how different countries regulate these drugs is missing. This study aimed to conduct a comparative analysis of the regulatory classification of drug commonly used for SM in children across ten countries. The goal was to uncover regulatory trends and variations based on key factors like product type and national economic level, providing a more nuanced understanding of this diverse regulatory landscape.

## Methods

This study employed a targeted review of regulatory documents, rather than a conventional systematic or scoping review. The aim was to compares regulatory classifications of common paediatric SM drugs across ten countries to uncover trends based on product type and national economic level.

Children were designated as people aged between 0 and under 18 years in accordance with the World Health Organization (WHO). They were further classified into four age brackets: infants (0–2 years), toddlers (2–5 years), middle childhood (6–11 years), and adolescents (12–17 years) (Hubal et al., 2014) to align with the International Council for Harmonisation of Technical Requirements for Pharmaceuticals for Human Use (ICH) and major regulatory agencies (ASEAN Secretariat, 2023; International Council for Harmonisation, 2024; Ministry of Health Pharmacy and Poison Board, 2022). This classification system reflects the significant physiological, metabolic, and developmental differences among these age groups.

The inclusion criteria for selecting the studied countries were based on a dual-criteria strategy. First, we focused on nations with stringent regulatory agencies as defined by the WHO (Australia, Canada, Japan, United Kingdom, and the United States of America) to identify robust standards for paediatric medicine classification (International Council for Harmonisation, 2024; Ministry of Health Pharmacy and Poison Board, 2022). Second, five ASEAN nations (Indonesia, Malaysia, the Philippines, Singapore, and Thailand) were selected based on our ability to secure reliable access to their official and comprehensive regulatory drug databases (ASEAN Secretariat, 2023).

Altogether, 12 drugs were selected based on two primary criteria. First, they had a documented high global frequency of SM, as evidenced by numerous prior studies (Bert et al., 2022; Gohar et al., 2017; Gualano et al., 2015; Italia et al., 2015; Pereira et al., 2007; Pfaffenbach et al., 2010; Shehnaz et al., 2014; Vernacchio et al., 2009; Ylinen et al., 2010). Second, they were commonly used drugs for treating minor illnesses and symptoms such as fever, pain, allergies, and cough. These conditions are widely promoted for SM practices worldwide. The selected drugs comprised those for treating common symptoms such as fever, pain, allergy, respiratory conditions (e.g. mucolytics and expectorants), asthma, and topical inflammation.

Primary data were extracted from the official drug regulatory websites of each chosen country. Supplementary data were sourced from national drug indexes, as elaborated in Table 1. Inclusion criteria for sources comprised: (1) public access, (2) regular updates, and (3) available data of drug classification status. Manual searches of official drug databases were conducted using a specific hierarchy. The initial search was performed using the brand name of the originator product (e.g. Tylenol) at a specified strength and dosage form. If the originator brand was not available in a database or had been withdrawn from the market, other common brand name was used. The active substance or generic name served as the final search option if no brand products were found. As drug classification status was retrieved from the official drug regulatory website, the information was regarded as highly accurate. However, drawing on insights from a study by Leelavanich et al. (2020) we paid close attention to cases where a drug's classification status might differ based on its package quantity or scope of its indications. This ensured our analysis captured the nuanced regulatory landscape of each country. To further guarantee the accuracy of data extraction, one researcher (RDF) primarily extracted information from the official national regulatory website, while another researcher (HA) independently conducted a separate search based on the product name specified by the RDF. The data collection duration was between June 2023 and February 2024. Data extraction concentrated on drug classification status, indication, and age suitability for paediatric use. Information was then delineated by specific drug, strength and dosage form across ten countries.

**Table 1.** Sources of drug classification information by country.

Country	Source	Reference
Australia (AUS)	Australian Register of Therapeutic Goods	https://www.tga.gov.au/resources/artg
		https://www.ebs.tga.gov.au/
Canada (CAN)	National Drug Schedules	https://www.napra.ca/national-drug-schedules/?keywords=sulindac&amp;schedule
	Drug Product Database online query	https://health-products.canada.ca/dpd-bdpp/index-eng.jsp
Indonesia (IDN)	The Indonesian Food and Drug Authority (BPOM)	https://cekbpom.pom.go.id/
Japan (JPN)	PMDA (Prescription)	https://www.pmda.go.jp/PmdaSearch/iyakuSearch/
	PMDA (OTC)	https://www.pmda.go.jp/PmdaSearch/otcSearch/
	Find OTC Medicines	https://search.jsm-db.info/sp_en/
Malaysia (MAL)	Official portal of pharmaceutical services programme	https://www.pharmacy.gov.my/v2/en/documents/poisons-list.html
	Industry and QUEST3+ System	https://quest3plus.bpfk.gov.my/pmo2/index.php
	MIMS Malaysia	https://www.mims.com/malaysia
The Philippines (PHP)	Human Drugs Products List	https://verification.fda.gov.ph/drug_productslist.php
Singapore (SGP)	Singapore Drug Database	https://pharmfair.com/
	Ministry of Health Singapore	https://www.healthhub.sg/programmes/hsa
Thailand (THA)	Drug Product Information search system	https://pertento.fda.moph.go.th/FDA_SEARCH_DRUG/SEARCH_DRUG/FRM_SEARCH_DRUG.aspx
The United Kingdom (UK)	MHRA Products	https://products.mhra.gov.uk/
	NHS UK	https://www.nhs.uk/medicines/#B
	Electronic Medicines	https://www.medicines.org.uk/emc/search?q=drug&t=advanced&st=true&sc=true&l=4
The United States of America (USA)	Drug@FDA	https://www.accessdata.fda.gov/scripts/cder/daf/index.cfm#drugName19
	National Drug Code Category	https://www.accessdata.fda.gov/scripts/cder/ndc/index.cfm

Note: the references list was cited from Leelavanich, et al. with some modifications (Leelavanich et al., 2020).

**Abbreviations**: MHRA (Medicines and Healthcare Products Regulatory Agency); NHS (National Health Service); PMDA (Pharmaceuticals and Medical Devices Agency); BPOM (Badan Pengawas Obat dan Makanan/The Indonesian Food and Drug Authority); MIMS (Monthly Index of Medical Specialties).

Two analytical methods were used: qualitative content analysis and comparative analysis. The former investigated trends in classification status across different dosage forms and age groups. The latter investigated both vertical (within-country) and horizontal (cross-country) comparisons among ASEAN and SRA countries, with a concentration on dosage forms, age segmentation, and regulatory classification.

To enable a meaningful cross-national comparison of drug regulations, we classified drugs using a validated four-tiered system for non-prescription which adapted from Leelavanich et al. (2020). This framework harmonises diverse national terminologies into consistent categories based on the level of regulatory control. In this study, drugs were classified as follow;

– Prescription drugs were labelled with ‘P’.
– Non-prescription drugs: Sub-categorised to reflect varying levels of access.
  • Behind-the-Counter (BTC): Drugs dispensed under the supervision of a pharmacist.
  • Open Shelf (OPS): Drugs that do not require pharmacy supervision but are sold in pharmacies or hospitals.
  • General Sale List (GSL): Drugs that can be sold outside of pharmacies, such as in convenience stores.
  • Transitional (T): Drugs available without a prescription under a specific regulatory exemption, as designated by the national authority.

**Figure 1.**
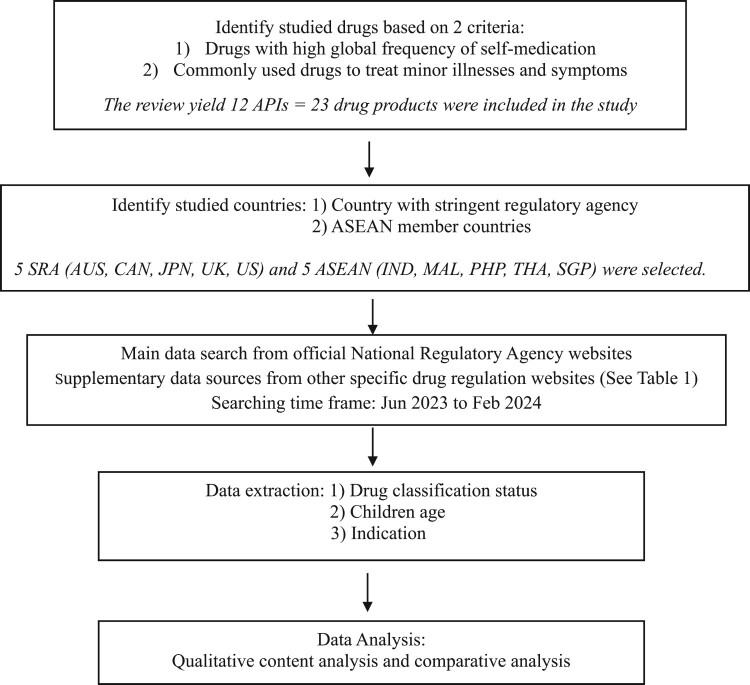
Flowchart of the methodology process.

Detail of drug classification system in each country is illustrated in Table 2. The study methodology is presented in a flowchart in Figure 1 for clarity and simplicity.

**Table 2.** Drug classification systems across countries.

Country	Prescription	Non-prescription
AUS	Prescription only medicines (Schedule 4)	Three sub-categories include: Pharmacist only medicines (Schedule 3) Pharmacy medicines (Schedule 2) Unscheduled (U)
CAN	Schedule 1	Three sub-categories include: Schedule 2 Schedule 3 Unscheduled
IDN	Prescription (OK)	Two sub-categories include: Limited medicine (OBT) Over the counter (OB)
JPN	Prescription	Four sub-categories include: Guidance mandatory drugs Type 1 non-prescription drugs Type 2 non-prescription drugs Type 3 non-prescription drugs
MAL	Group B	Two sub-categories include: Group C Non-poison
PHP	Dangerous drugs prescription (DD, Rx) Exempted dangerous drugs prescription (EDD, Rx) Prescription (Rx)	Two sub-categories include: Over the counter (OTC) Household remedies
SGP	Prescription only medicine (POM)	Two sub-categories include: Pharmacy only medicine (*P* Only) Over the counter (OTC)
THA	Specially controlled substance	Three sub-categories include: Dangerous drug (DD) Non-dangerous drug (NDD) Household remedies
UK	Prescription only medicine (POM)	Two sub-categories include: Pharmacy drug (*P*) General sale list (GSL)
USA	Prescription	Over the counter (OTC)

## Results

Table 3 presented the drug classification status for 12 active pharmaceutical ingredients (APIs), which included 23 specific generic drugs, strengths, and dosage forms, across five indications already identified as routinely applied in paediatric SM. A comparable classification pattern was seen for various drugs routinely applied for common colds.

**Table 3.** Status of drugs generally used for SM in children.

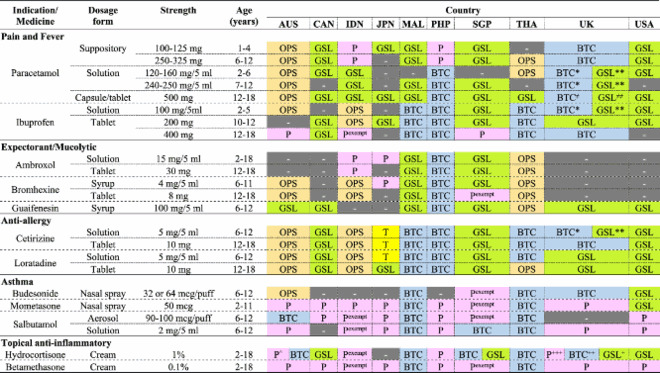

Notes: ‘-' medicinal products are not available in the country; Pink color refers to prescription drug; Blue color refers to behind-the-counter; Orange color refers to open shelf drug; Green color refers to general sale list; Grey color refers to product not available.

P^exempt^ refers to prescription only medicine with exemptions. The special status was found in Singapore and Indonesia.

*Volume > 100 ml, ** Volume ≤ 70 ml.

≠>16 tab/pack, ^≠≠^ refers to ≤ 16 tab/pack.

^+^ 10 gm/pack, ++ refers to ≤15 gm/pack, +++ refers to > 15 gm/pack.

^^^ 50 gm/pack, ^^ refers to 30 gm/pack.

When the unique drug products (defined by API, strength, and dosage form) were compared across the ten countries, most of those designated for pain and fever (e.g. paracetamol, ibuprofen), anti-allergy (e.g. cetirizine, loratadine), and mucolytic/expectorants (e.g. ambroxol, bromhexine, guaifenesin) were classified as ‘non-prescription'. As an example, paracetamol suppositories were classified as non-prescription in 8 out of 10 countries, and both paracetamol syrup and tablet forms were classified as non-prescription in all ten countries.

Remarkably, when exploring the same drug in different strengths or dosage forms targeted for older children, the classification by each country usually stayed unchanged. An example is, paracetamol in suppository, syrup, and tablet dosage forms which persisted to be classified as GSL in the USA, Canada, Japan, Australia, Singapore, and Malaysia, even as the strength rose. In addition, this pattern was also seen with ibuprofen, ambroxol, bromhexine, guaifenesin, cetirizine, and loratadine.

For the case of ibuprofen 400 mg tablets, which are normally applied in adolescents aged 12–18 years as well as adults, classification differences were noticed. For 3 out of the 10 observed countries, it was classified as ‘P’. Remarkably, Indonesia classified ibuprofen 400 mg with a unique status ‘prescription-only medicine with exemptions' (Pexempt). This classification permits pharmacists to dispense the drug without a prescription for specific emergency conditions. Nevertheless, that dispensing must be in compliance with strict criteria, comprising limited quantity of supply with mandatory documentation of patient information to support safety monitoring and follow-up.

Most countries classified drugs for asthma, including budesonide, mometasone, and salbutamol as ‘P’. For example, mometasone nasal spray was ‘P’ in 7 out of 10 countries. Remarkably, Singapore granted special access for mometasone nasal spray by classifying it as Pexempt. Additionally, salbutamol, in both aerosol and solution forms, was ‘P’ in most studied countries, with just a minority classifying it as a BTC category drug, which denotes limited availability for SM. These findings indicate a cautious regulatory attitude towards asthma drugs, which is probably because of their potential for misuse or delayed diagnosis of serious respiratory conditions.

With topical corticosteroids, classification status was mainly determined by drug potency. Hydrocortisone 1% cream, a low-potency steroid, was classified as a non-prescription drug in 7 out of 10 countries. Conversely, betamethasone 0.1%, a higher-potency corticosteroid, was classified as ‘P’ in 8 out of 10 countries.

Beyond potency, both pack size and approved indications also demonstrated key classification roles. As an example, in the UK hydrocortisone cream in 10 g size was classified as GSL, although it was classified as BTC or ‘P’ in higher volumes (e.g. 15 g or more than 15 g sizes, respectively). Moreover, regulatory status differed according to the indication. Hydrocortisone 1% approved for mild symptoms such as itching or irritation was often non-prescription, although the identical product labelled for conditions like dermatitis or eczema was limited to only prescription use. These findings have accentuated how national regulatory authorities combine multiple risk-based criteria, such as potency, quantity, and clinical indication, into classification decisions for topical steroids.

The analysis revealed clear patterns in drug classification based on risk. Lower-risk medications for pain and fever, such as Paracetamol and Ibuprofen, are predominantly classified as GSL or OPS across the studied countries, allowing broad public access. In contrast, drugs for asthma (Budesonide, Mometasone, and Salbutamol) were overwhelmingly classified as ‘P’. This reflected a global consensus that these potent, long-term medications required professional diagnosis and ongoing monitoring due to their risk profile. However, some countries like Thailand and the UK allowed Salbutamol to be BTC, while others, like Indonesia, Singapore, and the Philippines, offer Pexempt, highlighting a variation in risk tolerance. Similarly, topical corticosteroids demonstrated risk-based classification patterns influenced by potency and pack size. Hydrocortisone 1% cream was commonly classified as non-prescription in small pack sizes or for mild symptoms, whereas the same formulation was restricted to ‘P’ when indicated for chronic conditions such as eczema, or with higher volumes in a pack size.

A notable distinction in classification appeared to link to economic status. While high-income countries like Australia, Canada, Singapore, and the USA consistently classified non-sedative anti-allergy drugs as GSL or OPS, middle-income countries like Malaysia, the Philippines, and Thailand classified them as BTC. This suggested that middle-income countries may adopt a more restrictive regulatory approach to ensure a higher level of professional oversight for these widely used SM drugs.

## Discussion

To the best of our understanding, this is the first study to evaluate the drug classification status of commonly used drugs for children in SM practices across ten countries. This study refrained from assuming that drug regulatory agencies across countries would classify the same medicines in identical ways, because each country functions within a distinct context. Additional to drug efficacy and safety profiles, variations in national socioeconomic status, compulsory education, health literacy, availability of healthcare services, and provision of public health insurance all impact regulatory decisions. Furthermore, the frameworks for classifying non-prescription drugs vary across countries. Regardless of these variations, this study found remarkable similarities in how drugs for five common paediatric symptoms are regulated across both high-income and upper-middle-income countries which indicated shared orientation in balancing availability with safeguards.

When compared with a prior study (Leelavanich et al., 2020), that evaluated the classification of drugs applied in adult SM, only one product; cetirizine syrup, overlapped with this current study. Comparing the two studies demonstrated that three countries had changed the drug classification status of cetirizine syrup to a less restrictive category. Particularly, in the United States, cetirizine syrup was reclassified from ‘P’ to GSL; in Canada, from OPS drug to GSL; and in Singapore, from BTC to GSL. These changes may represent growing trust in cetirizine’s safety profile, enabling wider availability in both adults and children.

Drug classification indicated a regulator’s perspective on either empowering the public to be responsible for treating minor illnesses or enforcing stricter regulations that transfers responsibility from the general public to healthcare professionals. Those decisions need careful balancing between enabling availability to certain drugs and ensuring that people have enough health literacy to use them befittingly. Moreover, regulatory agencies may combine multiple risk-based criteria, such as drug strength, potency, quantity, and approved clinical indications, into their drug classification decision making. This more refined method enables policies to better reflect patients’ requirements while safeguarding public health.

Drug classification solely may not completely indicate consumer availability. Regulators can enact supplementary measures to enhance availability without modifying a drug’s legal status. As an example, in Singapore and Indonesia, some drugs remain ‘P’ but can still be dispensed without a prescription under specific protocols. These comprise pharmacist supervision, quantity limits, and mandatory patient usage documenting to promote safe and suitable use, balancing availability with public health safeguards.

One notable issue highlighted by this study is that SM with ‘P’ in children such as inhaled budesonide, mometasone, or salbutamol heightens significant matters. Due to their potent nature and the need for personalised dosing and ongoing monitoring, asthma medications are almost universally categorised as ‘P’. However, since asthma is a chronic condition, patients often use existing medications to manage sudden flare-ups after an initial diagnosis. This practice highlights a tension between the need for a strict regulatory status and the desire for patients to have timely access to essential care. Regulatory bodies could address this by considering new classifications, such as a Pexempt status for certain drugs during an emergency. Alternatively, without creating a new status, supplemental measures could be implemented to ensure safer SM practices. These could include providing comprehensive patient information leaflets (PILs) or establishing personalised emergency management plans for flare-ups, which would allow patients to safely manage their condition at home.

This study has several limitations. First, it relied solely on document analysis using publicly available records, regulations, and publications, without direct engagement from stakeholders. While this method supported broad cross-country comparisons, it might have lacked contextual nuance and in-depth understanding of implementation challenges that qualitative data can provide. Therefore, our findings reflect the written policy but may not fully capture the underlying rationale behind certain regulatory decisions. Future research should incorporate additional design such as semi-structured interviews with experts and regulatory officials. This approach would complement our findings by providing a richer understanding of the decision-making processes, implementation barriers, and stakeholder perspectives on regulatory policies. Second, data were collected during a single period in time (June 2023–February 2024), which captured just a snapshot of the current classification landscape. Drug regulatory statuses are subject to change post data collection. Furthermore, a drug's official regulatory status may not directly equate to its practical availability to consumers, especially through less regulated channels such as online markets. Follow-up studies may be required to monitor ongoing policy changes and their implications. A third limitation is that the availability of branded drugs varies across countries, which may introduce a potential for bias. This is especially true in countries where a drug's classification status depends on its specific brand name rather than its API. Lastly, a key limitation of our study is the use of a harmonised drug classification scheme. While this system is valuable for comparing ‘P’, BTC, OPS, and GSL drugs across countries, it may not capture the full complexity of each nation’s regulations. These distinctions can be significant and are often influenced by factors such as the paediatric patient's age, specific indications for use, package quantity, or special regulatory exemptions.

While drug classification concepts normally remain consistent, specific drug categories differ across countries. Many factors affect each nation's drug classification, including adverse event reports, increased market potential for non-prescription drugs, impact of generic competition on over-the-counter (OTC) trade after patent expiry, and how easily available non-prescription drugs are (Gauld et al., 2019; Sukrod, 2015). Therefore, drug statuses do not remain static; they frequently change. Those reclassifications can stem from changes in professional medical paradigms, evolving health awareness, or improvements in a medicine's safety profile (Leelavanich et al., 2020). Remarkably, although children metabolise drugs differently from adults, their SM rates are comparable to adults (Stephenson, 2005).

The effective treatment of paediatric patients requires a meticulous approach to drug and prescription management throughout the entire drug administration process, with potential challenges coming from physical and social differences. The diverse spectrum of ages, weights, and body surface areas of paediatric patients requires precise calculations for individualised dosages (Stephenson, 2005). Additionally, the limited supply of appropriate formulations for this demographic often necessitates pharmacists to promptly compound specific products (Ackers et al., 2007). Furthermore, the participation of caregivers in the treatment process emphasises the value of maintaining prescription status for medications (Bi et al., 2023; Stephenson, 2005). This ensures that caregivers are given comprehensive education on suitable, potential adverse effects specific to children, and proper methods for safely storing medications (Benavides et al., 2011).

Several countries such as the UK, Singapore, Indonesia, and the Philippines had differing regulations for the identical active ingredients marketed under different brand names, which resulted in different statuses. Meanwhile, countries such as the USA, Canada, Japan, Australia, Malaysia, and Thailand maintained the same status for an active ingredient, regardless of its brand name, such as ‘generic name + strength + dosage form + pack size' were granted the same drug status. Those inconsistencies can affect key stakeholders in the drug classification system, although the drug regulatory agencies are the only entity to ultimately approve this decision.

This approach of maintaining the same status will assist in standardising drug information across brands, thereby simplifying understanding and control of drug statuses. By generalising drug status information, it will become appreciably easier for medical and regulatory personnel to manage and communicate this information. Clear and consistent drug statuses assist in maintaining an organised and efficient healthcare system, ultimately improving patient safety and care. When a status is downgraded, suitable strategies are essential to ensure its safe and effective use, such as a stricter regulation for the volume; adjustment of pack sizes and number of tablets; using patient information leaflets and other medias to guide in proper usage and adherence.

Concerning the drug classification system, a T drug group in Japan permitted patients to access T drugs without a prescription through pharmacist education (Nomura et al., 2016). These transitional drugs can be reclassified to a lower status, if the drug regulatory agency and pharmaceutical industries supply enough safety information for further use. Singapore and Indonesia have a special system which is called Pexempt which permits healthcare workers to supply prescription drugs without a prescription, if patients satisfy specific criteria, such as indication, strength, maximum daily dose, supply limits, and age restrictions (Health Sciences Authority, 2024a, 2024b). These medicines can be candidates for reclassification, either to a higher or lower status. As an example, ibuprofen was reclassified from ‘P’ to GSL in Singapore.

To ensure patient safety, stakeholders develop strategies to support suitable administration and safety information to increase patients’ literacy. For example, the information labelled on drug packaging should be clear and concise, especially for the particular population targeted by the products, and especially for the age range of children along with their suitable dose per day (in mL) should be in exact and easy numbers for caregivers to apply. Furthermore, some warnings and precautions associated with the limited condition of treatment, such as ‘do not use more than four times per day’ or ‘do not use more than 2 days’ or ‘do not administer to children under 3 years old’, are the strategies used to educate patients when performing SM to avoid mistaken dosage and direction to use. Education through the patient information leaflet should help patients to read consecutively and to carefully follow the directions of use. In addition, proper pack sizes and packaging are designed by the manufacturers to prevent misuse and abuse by giving the exact volume of drugs for patients’ needs. For instance, the front of the package showed the brand name (Advil®), actual active ingredient (ibuprofen), dosage form (suspension), strength (100 mg/5 ml), and packaging volume (120 ml) (U.S. Food and Drug Administration, 2022).

In response to the paediatric medication policy, a suggestion has been made. Some of the most frequently prescribed drugs applied for SM in children should be available as non-prescription drugs, but only in certain most common dosage forms which are suitable for the age of the children. Another suggestion is that pharmaceutical companies could create a new strategy to ensure the safety of patients when using paediatric drugs. To generate a new strategy, health regulation authorities should act on the issues where the most frequently used SM drugs in children were purchased inappropriately and review different approaches to control. In addition, pharmaceutical companies and health regulation authorities can collaborate on marketing authorisation for new paediatric medicines to change from ‘P’ to nonprescription in a short time after they are released.

Recommendation for further implementation should focus on establishing Advocacy Groups for drug review. Those groups would be responsible for the regular review and evaluation of the current list of drugs before considering status changes. In addition, further research is crucial to gain a comprehensive understanding of the underlying reasons and motivations for paediatric SM, which should include in-depth interviews. Those interviews can provide qualitative insights by allowing participants, especially drug regulatory personnel, to express their perspectives, experiences, and explanations in detail, offering a richer context beyond what is available in written documents. These recommendations will enable efficient management of the transition of drug statuses, ensuring that both healthcare providers and patients are well-informed and able to adapt to changes in drug access and regulation.

## Conclusion

Drugs routinely applied for SM in children are classified as prescription and non-prescription. Whereas these drugs share the same active ingredients, dosage forms, and strengths, their regulatory status often differs across countries. This lack of harmonisation underscores a critical need for a globally consistent paediatric drug classification scheme to ensure safety and clarity. Nevertheless, the most crucial priority is ensuring SM in paediatric populations is properly performed. Our results suggest that regulatory agencies have a key role to play not only in setting drug classifications but also in empowering caregivers and mature adolescents who can responsibly manage their own health.

By providing clear and accessible patient information leaflets and other educational initiatives, regulators can enable the public to use essential medicines safely and appropriately, ultimately balancing public health safeguards with access to care.

## Data Availability

The study relied on publicly available information that is legally accessible.
